# Nonpodocyte Roles of APOL1 Variants: An Evolving Paradigm

**DOI:** 10.34067/KID.0000000000000216

**Published:** 2023-09-28

**Authors:** John Pell, Soichiro Nagata, Madhav C. Menon

**Affiliations:** Department of Medicine, Yale University, New Haven, Connecticut

**Keywords:** genetic renal disease, glomerulopathy, kidney transplantation

## Abstract

Since the seminal discovery of the trypanolytic, exonic variants in apolipoprotein L1 (APOL1) and their association with kidney disease in individuals of recent African ancestry, a wide body of research has emerged offering key insights into the mechanisms of disease. Importantly, the podocyte has become a focal point for our understanding of how risk genotype leads to disease, with activation of putative signaling pathways within the podocyte identified as playing a causal role in podocytopathy, FSGS, and progressive renal failure. However, the complete mechanism of genotype-to-phenotype progression remains incompletely understood in APOL1-risk individuals. An emerging body of evidence reports more than podocyte-intrinsic expression of APOL1 risk variants is needed for disease to manifest. This article reviews the seminal data and reports which placed the podocyte at the center of our understanding of APOL1-FSGS, as well as the evident shortcomings of this podocentric paradigm. We examine existing evidence for environmental and genetic factors that may influence disease, drawing from both clinical data and APOL1's fundamental role as an immune response gene. We also review the current body of data for APOL1's impact on nonpodocyte cells, including endothelial cells, the placenta, and immune cells in both a transplant and native setting. Finally, we discuss the implications of these emerging data and how the paradigm of disease might evolve as a result.

## African Ancestry, Kidney Disease, and APOL1 Variants

CKD presents an ongoing challenge in public health, with nondialysis patients with CKD accounting for 18.2% of total Medicare expenditures and individual patients accumulating $22,348/yr in medical costs—approximately 3 times as much as non-CKD patients.^1^ In the United States, individuals with recent African ancestry (AAs) have been consistently reported as experiencing an increased incidence of kidney disease when compared with Caucasians, with a 3.5-fold higher rate of ESKD.^2^ The risk of CKD/ESKD among AAs shows familial clustering that is not completely accounted for by hypertension, diabetes mellitus, or socioeconomic status, indicating a role for genetic factors in increased disease risk.^3,4^ Among etiologies of renal diseases among AAs with CKD, FSGS,^5^ lupus nephritis (LN),^6^ sickle cell nephropathy,^7^ and hypertensive nephrosclerosis^8^ have been repeatedly associated, whereas the association with diabetic nephropathy as a cause of progressive CKD among AAs is inconsistent.^9^ These data suggest that specific pathogenetic mechanisms, rather than nonspecific progression of CKD, likely underlie the increased risk of CKD/ESKD among AAs.

In 2010, Genovese *et al.* made the seminal discovery that the presence of two mutually exclusive exonic variants of the gene encoding the trypanolytic factor apolipoprotein L1 (APOL1) on chromosome 22—G1, a missense variant, and G2, a six-base pair deletion, correlated with a ten-fold higher risk for FSGS/ESKD in individuals carrying these risk genotypes (versus the wild type variant, G0).^10^ Adjusting for other genetic variants within chromosome 22 did not eliminate APOL1 variant association with kidney disease, whereas adjusting for APOL1 variants failed to produce any other disease associations. Thus, APOL1 risk genotype nearly completely accounted for the observed risk of progressive CKD among AAs.

Here, we review the reported paradigm and mechanisms for APOL1-mediated kidney disease and discuss the apparent shortcomings of this paradigm regarding clinically observed APOL1 disease. We examine emerging data which highlight extra-podocyte roles of G1-/G2-APOL1 that potentially contribute to disease progression in risk-genotype individuals and help illuminate present gaps in the epidemiology and mechanism.

## The Podocentric Paradigm: Mechanisms, Inconsistencies, Shortcomings

Aside from humans, APOL1 is only expressed in old-world monkeys and some apes,^11^ slowing mechanistic studies that rely on rodent models. In these species, APOL1 is a component of HDL molecule and is expressed in many organs such as the liver, lung, placenta, brain, and kidney. APOL1, the only secreted member of the APOL family of proteins owing to the presence of a signal peptide, circulates in the blood where its principal trypanolytic role has been described.^12^ The main source of this circulating APOL1 is the liver. Secreted as a part of the HDL3 molecule, APOL1 G0 is taken by *Trypanosoma Brucei* (*T. Brucei*) subspecies *Brucei via* endocytosis and inserts itself on the endosomal membrane because of its membrane-addressing domain. Under acidic conditions of the trypanosomal lysosomes, it functions as a cation channel on the membrane causing ion influx, lysosomal swelling, and cell death of *T. Brucei*. In addition, APOL1 contains Bcl2-homology 3 domain suggesting a role for this protein in autophagic cell death.^13^ Distinct from *T. Brucei*, in *T. Brucei* subspecies *rhodesiense*,^14^ APOL1 G0 colocalizes with serum resistance-associated (SRA) protein whose expression in this subspecies and binding to APOL1 G0 significantly inhibited the channel activity.^15^ Conversely, APOL1 G1 and G2 variants, which emerged in populations of recent African ancestry, are defined by mutations altering the SRA-binding domain of APOL1 G0, allowing these to avoid binding with parasitic SRA and, therefore, kill *T. Brucei* rhodesiense, together representing a coevolutionary arms race between parasite and host.^12^

The interest in a role for APOL1 G1 and G2 variants in podocytes emerged with identification of their association with FSGS and progressive CKD in AAs. In 2017, Beckerman *et al.* first reported that risk genotypes overexpressed in podocytes play a crucial role in podocyte injury and that risk-genotype expression correlated with disease severity, proposing the threshold hypothesis.^16^

Many studies have since reported possible mechanisms of APOL1 variant expression-related podocyte injury in cells, such as cation channel conductance, mitochondrial dysfunction, endoplasmic reticulum (ER) stress, pyroptosis, global suppression of protein synthesis, dysfunction of ubiquitin-proteasome systems, and autophagic block, among others.^17^ In drosophila, Gerstner *et al.* expressed APOL1 in podocyte-like nephrocytes, demonstrating that risk variants elevated endocytic function in these cells and increased signs of early cell death.^18^ G1-/G2-APOL1–expressing drosophila nephrocytes demonstrated phenotypes suggesting an ER stress response, that is, ER swelling and chaperone induction. In tubular cells, Shah *et al.* reported APOL1 protein translocation to the mitochondrial matrix, and G1-/G2-APOL1 formed higher-order oligomers versus G0-APOL1 and increased mitochondrial permeability transition pore mitochondrial pore opening—suggesting a pathway with mitochondrial pore opening as the first step to cell death.^19^ However, mitochondrial translocation of APOL1 proteins was not observed in the drosophila study.

A major factor contributing to the complexity of mechanistic studies is that because rodents lack the APOL1 gene, most have used overexpression models, making it difficult to exclude artifacts of overexpression from their findings. As a more physiological model, McCarthy *et al.* reported that risk-genotype bacterial artificial chromosome-transgenic mice showed significant albuminuria *via* IFN-*γ*–expressing plasmid injection by tail vein.^20^ Nystrom *et al.* showed that a combination of IFN-*γ* and IFN-*α*/*β* are necessary to significantly increase APOL1 levels in human kidney organoids.^21^ Interestingly, Janus kinase-signal transducer and activator of transcription inhibitors completely suppressed APOL1 and rescued cell viability.

Recently, Egbuna *et al.* reported that inaxaplin, an APOL1 channel function inhibitor, reduced proteinuria by 47.6% after 13 weeks of treatment in a phase 2a clinical study,^22^ supporting the hypothesis of APOL1 as a cation channel. Although they lack a comparison with a control group and how inaxaplin affects podocytes was not clarified, these results provide urgent motivation to study this mechanism. It is well-known that APOL1 functions as a cation channel to kill *T. Brucei via* pore formation in endosomal, mitochondrial, and plasma membranes. Furthermore, differential effects of G1-/G2-APOL1 on *T. Brucei* Gambiense are due to resistance of these risk variants against neutralization by SRA factors produced by this subspecies, hence allowing cation-channel activity in parasitic lysosomes in risk-genotype individuals. Indeed, Madhavan *et al.* reported that APOL1 colocalizes with vesicle soluble N-ethylmaleimide-sensitive factor attachment proteins receptor vesicle-associated membrane protein, yet G1-/G2-APOL1 fails to bind to vesicle-associated membrane protein, possibly leading to kidney disease.^23^ Hence, although translationally relevant data have been reported, many key cellular mechanisms of APOL1-FSGS remain nebulous.

### Shortcomings of the Podocentric Paradigm: the Need for a Second Hit

Although these data clearly insinuate that podocyte expression of G1-/G2-APOL1 and engagement of downstream signaling mechanisms is necessary for pathogenesis, the podocentric paradigm has its shortcomings. Lifetime risk for ESKD in humans with two RVs was shown to be only approximately 15%^24^—that is, most RV genotype individuals will not develop progressive CKD. In single cell RNA sequencing data interrogating the human kidney, homeostatic podocytes show high APOL1-mRNA expression versus other kidney cells.^25,26^ If podocyte-intrinsic G1-/G2-APOL1 expression was sufficient to drive the disease, the lifetime risk of APOL1-FSGS ought to be significantly higher. Furthermore, a constitutive podocyte-specific APOL1-expression mouse model (using Nphs1promoter) did not induce overt kidney disease or albuminuria in spite of reduced podocyte density.^27^ The authors speculated that a combination of podocyte G1-/G2-APOL1 expression, reduced podocyte density, and another upstream hit is required to drive APOL1-mediated FSGS.

Indeed, APOL1-mediated kidney disease has been stereotypically linked to several infectious triggers in epidemiologic studies and clinical reports. APOL1-FSGS is strongly associated with HIV-associated nephropathy,^28^ parvovirus infection,^29^ and cytomegalovirus infection in transplant recipients.^30^ Recently, coronavirus disease 2019 (COVID-19) emerged as a trigger for G1-/G2-APOL1–induced collapsing glomerulopathy in native and allograft kidneys.^31,32^ Early in the COVID-19 pandemic, two case studies were reported in which RV-expressing patients who both tested positive for COVID-19 infection were found to have symptoms of FSGS after biopsy and the presence of viral particles in the proximal tubule epithelium cytoplasm and in podocytes.^33^ In these cases of infection-associated FSGS, treatments directed toward the infection (highly active antiretroviral therapy, anti–COVID-19 therapy) were reported to benefit the disease,^32,34^ supporting a role for treatable disease modifiers. Recently, Wu *et al.* investigated histopathologic, genetic, and molecular features in six Black patients with COVID-19 infection, AKI, and *de novo* nephrotic range proteinuria.^35^ The authors reported an association between collapsing glomerulopathy and G1-/G2-APOL1 genotype, and interestingly, an absence of direct viral infection in the kidneys—contrary to the findings of previous case studies. The authors discussed that their data supported the presence of a two-hit mechanism in APOL1-FSGS and/or an extra-podocyte effect for the APOL1 protein in response to a viral or inflammatory trigger.

Existing literature could also mechanistically connect viral infection (or an inflammatory process) to the development of disease in risk-genotype AAs. For instance, APOL1 itself was first identified as a highly cytokine-responsive gene.^36^ Correspondingly, the APOL1 promoter has binding sequences for STAT2 and IFN-responsive transcription factors—signals downstream of IFNs. Innate or adaptive immune cells are primarily activated and respond to infection by generating a milieu of cytokines—principal among which are IFNs (type-1/type-II).^37,38^ Analogously, SLE and LN are well-understood states of IFN excess,^39–41^ and stigmata of IFN excess—tubuloreticular inclusions in glomerular endothelial cells—are typically associated with APOL1-FSGS. Increased release of IFNs from APOL1 variant–expressing podocytes *via* activation of nucleoside sensor, stimulator of IFN genes (STING), and the NLRP3 inflammasome has also been observed.^42^ The STING-IFN pathway was also implicated in APOL1-related collapsing FSGS from case reports of patients with genetically overactive STING-IFN signaling.^43,44^ Specifically, direct IFN-*γ* (IFNG) stimulation in cultured podocytes increased G1/G2-APOL1 levels up to 12-fold and up to 200-fold in endothelial cells.^45^ IFNG expression was found to result in upregulation of APOL1 protein expression with robust induction of heavy proteinuria and FSGS in variant transgenic mice, but not in controls.^20^ These data suggest that although homeostatic podocyte RV-APOL1 expression alone may not drive disease, underlying infectious/immune stimuli that may act as triggers by upregulating podocyte APOL1 *via* IFNs, thus acting as second hits that modify disease risk and should, therefore, be a focus of research.

### Genetic and Environmental Factors as Third Hits

Although viral infection is established as a stereotypic trigger for APOL1-FSGS, not every G1-/G2-expressing individual who encounters viral insult develops disease—additional genetic and/or environmental modifiers or “third hits” likely play a role to modify disease risk.

To evaluate the presence of non-APOL1 genetic modifiers, Langefeld *et al.* performed genome-wide association studies and meta-analyses on 2650 patients with ESKD and 1656 controls.^46^ No gene associations with ESKD beyond APOL1 were detected, and although the authors conceded that variants with minor effects could not be ruled out, they concluded that environmental factors may be more important in modifying disease risk than genetic causes. Moreover, an intriguing report found that among pregnant APOL1 patients in the Boston area, risk-genotype individuals of AA ancestry born within the United States were at greater risk for preeclampsia versus those born outside of the United States but migrated, possibly suggesting the importance of environmental modifiers over AA ancestry–associated genetic loci.^47^ Conversely, a recent report demonstrated a significant association between APOL1-mediated kidney disease and missense variants in the inflammasome pathway by subjecting all identified variants to gene-set enrichment analysis, suggesting the presence of some genetic modifiers in mechanisms of disease.^48^ Moreover, this finding lends further credence to the finding that the NLRP3 inflammasome pathway plays a central role in the APOL1-FSGS disease mechanism.

## Nonpodocyte APOL1

**Figure 1:**
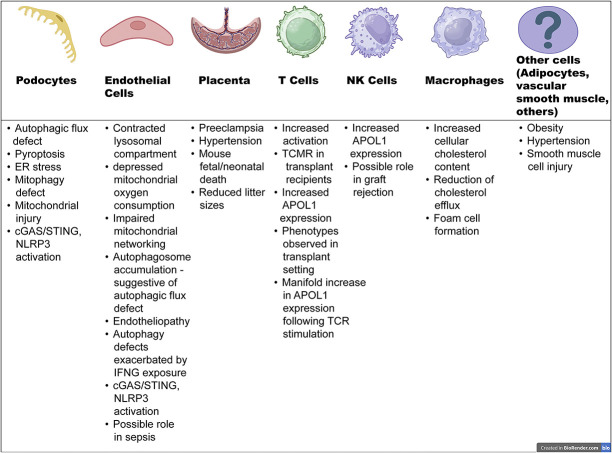
**Schematic shows proposed roles of APOL1 in podocyte and nonpodocyte cells.** APOL1, apolipoprotein L1; ER, endoplasmic reticulum; STING, stimulator of IFN genes.

Consistent with clinical observations described above, there is a growing body of data for extra-podocyte roles of APOL1 risk variants. Evolutionarily, the entire APOL gene cluster shows evidence of positive selection during primate evolution, with APOL1 itself making its appearance approximately 30 million years ago.^49^ Among this gene family, APOL1 is the only secreted protein.^50^ As described above, the primary understanding of its immune response role in humans comes from the trypanolytic function of secreted APOL1, which is taken up by the parasite as part of the HDL3 molecule.^36^ In the low pH environment of the parasite lysosome, APOL1 inserts into the membrane and induces ion influx causing lysosomal swelling and cell death, serving as an innate immune mechanism to neutralize *T. Brucei*. Crucially, G1/G2 variants, but not G0, neutralize *T. Brucei* Rhodesiense.^11^ These established functions of APOL1, along with aforementioned data, suggest a primary role for this gene cluster and specifically APOL1 as an antimicrobial immune response gene, potentially providing survival advantage to the host during one or more microbial infection. Thus, these point to fundamental nonpodocyte roles for APOL1 (Figure 1).

### Endothelial Cells

Blazer *et al.* evaluated G1-/G2-APOL1's impact on endothelial cells in human umbilical vein endothelial cell (HUVEC) cultures, comparing different APOL1 genotype HUVECs both at baseline and in response to treatment to IFNG.^51^ The authors reported that IFNG increased APOL1 expression across all genotypes 22.1-fold; however, two RV copy-carrying HUVECs showed depressed baseline/maximum mitochondrial oxygen consumption and impaired mitochondrial networking on mitotracker assays. Thus, APOL1 RVs appear to have a cytotoxic effect on endothelial cells. G1-/G2-APOL1–expressing HUVECs demonstrated a contracted lysosomal compartment and autophagosome accumulation, suggesting an autophagic flux defect (as also shown in murine podocytes by Beckerman *et al.*). Compared with HUVECs carrying 0- or 2-risk variant copies, one-copy HUVECs demonstrated intermediate mitochondrial respiration and autophagic flux phenotypes that were exacerbated with IFNG exposure, suggesting an allele dose-dependent effect. This finding represents the first description of pathobiology in cell cultures from heterozygous G1-/G2-APOL1, supporting an additive model of disease risk over the traditional recessive model.

Recently, Wu *et al.* reported data from single-cell analysis performed on human kidney samples, revealing an association between G1-/G2-APOL1 and COVID-19 severity as well as endothelial cell defects which exacerbated sepsis.^52^ The authors evaluated data from 57,000 Black patients in the million veteran program program. They found that risk variant genotype associated with AKI in patients with COVID-19, as well as septicemia diagnosis. The authors then analyzed plasma samples from 70 Black patients in the cohort of intensive care patients, reporting that plasma APOL1 levels correlated with AKI development and severity as well as overall 30-day mortality.

On the basis of these findings, the authors performed molecular profiling on mice with endothelial cell specific G1-/G2-APOL1 expression (endothelial cell [EC]/G0APOL1, EC/G2APOL1), revealing a correlation between G1 /G2 genotype and several markers of endotheliopathy, including vascular inflammation, permeability, adhesion molecules, and endothelial glycocalyx remodeling. In both LPS and cecal perforation models of sepsis, severity was higher in EC/G2APOL1 mice with increases in genetic markers of inflammation and circulating cytokines. Interestingly, the authors also found mitochondrial defects that drove cyclic GMP–AMP synthase/STING and NLRP3 inflammasome activation, which when either genetically or pharmacologically inhibited, ameliorated endotheliopathy and sepsis in EC/G2APOL1 mice—highlighting the same mechanisms of injury as observed in podocytes.

### Placental Cells

Bruggeman *et al.* used a constitutive podocyte-specific APOL1 expression (Nphs1-promoter driven) and observed significantly increased severe pregnancy-related phenotypes in female mice, including preeclampsia, eclampsia, fetal/neonatal death, and small litter sizes.^27^ Although this could reflect a phenotype driven by podocyte-specific variant APOL1 expression including podocyte or nephron loss, nephrin is also expressed in placental trophoblast cells for syncytialization, and Nphs1-driven APOL1 variant expression in the placenta may have played a role as a driver of this phenotype.^53,54^ Analogously, APOL1 risk genotype has been linked to preeclampsia in human studies, as first demonstrated by Reidy *et al.* in a clinical study where fetal, but not maternal, APOL1 genotype associated with preeclampsia risk.^55^ These findings were recapitulated by Miller *et al.* in a study involving Black women from a single center in Ohio which included gross/histopathologic evaluations of placental tissues.^56^ However, the two studies did not reach a consensus on the mode of inheritance for the risk alleles, and the combination of environmental and genetic factors that factor into disease risk has not yet been fully elucidated.

### Obesity and Hypertension

Nadkarni *et al.* reported an association between G1-/G2-APOL1 and obesity/body composition.^57^ Among 11,930 self-reported AAs, the authors found that individuals with two RV-APOL1 alleles have 30% higher odds of obesity. Interestingly, an additive model was the best fit for the association, with each risk allele increasing obesity odds by 1.13-fold and increases BMI by 0.36 kg/m^2^, again suggesting that every copy of RV-APOL1 may contribute to this phenotype. An additive model of risk variant association with systolic BP and hypertension has also been reported,^58^ with AAs in the 20–29-year age range showing an increase in systolic BP of 0.94±0.44 mm Hg per risk variant copy and 2–5-year decrease in age at diagnosis of hypertension among APOL1 variant allele homozygotes.

A possible mechanism for the latter association involves vascular smooth muscle cells, which have been reported to contribute to APOL1-induced podocyte injury in an HIV milieu.^59^ Human umbilical artery smooth muscle cells overexpressing G1-/G2-APOL1 showed several-fold greater injury in response to treatment with conditioned media of HIV-infected PBMCs, U939 cells, or recombinant IFNG versus G0 controls. The authors conclude that human umbilical artery smooth muscle cells could serve as an endocrine or paracrine source of APOL1. Interestingly, Madhavan *et al.* reported localization of APOL1 to a subset of *α*-smooth muscle actin-positive cells in FSGS and HIV associated nephropathy but not normal kidney samples,^60^ further suggesting a role for APOL1 in smooth muscle cells.

### Immune Cells

The rapid and positive evolutionary selection of APOL group of genes,^49^ its high expression on cellular stimulation by cytokines, and trypanolytic function all support a role for APOL1 as an immune response gene, a characterization that could also explain associations between viral diseases, APOL1 overexpression, and FSGS. In a transplant setting, we reported evidence of an extra-podocyte role for APOL1. *Ex vivo* studies of PBMCs from APOL1-genotyped transplant recipients revealed high expression levels of APOL1 in activated CD4^+^/CD8^+^ T cells and natural killer cells. Interestingly, naïve CD4^+^/CD8^+^ T cells that had low APOL1 expression increased APOL1-mRNA levels manifold after T-cell receptor stimulation, possibly suggesting a role in activated T-cell responses. Single-cell transcriptomics in healthy controls and pretransplant waitlisted individuals revealed enriched activation signatures among variant-expressing T cells. At the protein level, IFNG-ELISPOT against a panel of donor B cells was increased with G1 or G2 versus G0. Clinically, these translated to an increased post-transplant T-cell–mediated rejection and reduced graft survival in recipients with high-risk genotype grafts, independent of donor genotype.^61^ Interestingly, this risk was observable in an additive model, namely, each copy of a risk variant increased risk. An additive model for disease risk has also been supported in the context of LN, where it has been reported that a single APOL1-RV copy increased risk among patients with LN for advanced CKD.^62^

A role for G1-/G2-APOL1 in macrophage function by promoting foam cell formation has also been reported.^63^ G1-/G2-expressing M1 macrophages had increased cellular cholesterol compared with wild type/G0 controls, although this phenotype was only seen in G1-APOL1 M2 macrophages and not G2s. There was also an observed reduction of cholesterol efflux as well as mRNA expression of cholesterol transporter genes ABCA1 and ABCG1 in G1-/G2-expressing macrophages. Clinically, the authors reported a positive correlation in patients expressing two risk alleles between APOL1 risk allele mRNA expression and genes involved in cholesterol efflux, thus positing a role for APOL1 risk variants in impairing reverse cholesterol transport in macrophages, which could potentially drive inflammation in the glomerulus and renal interstitium.

## Summary

Since the discovery that G1-/G2-APOL1 alleles underlie the disproportionate risk of progressive CKD and FSGS in AAs, research has led to key insights into disease mechanisms. The podocentric paradigm provides a compelling case for APOL1 within the kidney, with general agreement regarding podocyte APOL1 variant expression as likely necessary for injury. Significant progress has also been made in elucidating multiple putative mechanisms initiated by gene expression that could lead to podocyte injury. However, a unified picture to explain observed epidemiology, such as why only a limited percentage of people with two risk alleles develop kidney disease and disease occurrence in AAs, has not fully emerged including critical second/third hits. This, along with nonkidney phenotypes associated with APOL1-RVs, points to the need to consider a broader paradigm that incorporates nonpodocyte roles of APOL1, including within the immune system.

Additional studies are needed at the basic and clinical levels before a comprehensive mechanism of APOL1-mediated kidney disease, a critical public health problem, is unraveled.
